# A longitudinal study of cannabis use increasing the use of asthma medication in young Norwegian adults

**DOI:** 10.1186/s12890-019-0814-x

**Published:** 2019-02-26

**Authors:** Jørgen G. Bramness, Tilmann von Soest

**Affiliations:** 1 Norwegian National Advisory Unit on Concurrent Substance Abuse and Mental Health Disorders, Innlandet Hospital Trust, P.O. Box 104, 2381 Brumunddal, Hamar, Norway; 2 Institute of Clinical Medicine, University of Tromsø – The Arctic University of Norway, Tromsø, Norway; 3 Department of Psychology, University of Oslo, Oslo, Norway; 4 Norwegian Social Research, OsloMet – Oslo Metropolitan University, Oslo, Norway

**Keywords:** Adolescent, Cannabis, Asthma medication, Longitudinal, Smoking

## Abstract

**Background:**

A small number of studies have shown that the use of cannabis increases the risk of bronchial asthma. There is, however, a paucity of longitudinal studies which are able to control for known risk factors of bronchial asthma.

**Methods:**

Survey data from a population-based longitudinal study encompassing 2602 young adults followed for 13 years were coupled with individual prescription data on asthma medication (β_2_-adrenergic receptor agonists and glucocorticoids for inhalation) from the Norwegian national prescription database, which covers the entire Norwegian population. Current cannabis use, gender, age, years of education, body mass index (BMI; kg/m^2^) and current smoking were measured.

**Results:**

Prescription of asthma medication was associated with female gender, self-reported earlier asthma and allergies, daily tobacco smoking and current cannabis use. In a model adjusting for gender, age, years of education, BMI, earlier self-reported asthma and allergies and current tobacco smoking the odds ratio for a current cannabis user to fill prescriptions for asthma medication was 1.71 (95% CI: 1.06–2.77; *p* = 0.028).

**Conclusions:**

This suggests that cannabis is a risk factor for bronchial asthma or use of asthma medication even when known risk factors are taken into consideration. Intake of cannabis through smoking should be avoided in persons at risk.

## Background

Bronchial asthma is a common long-term inflammatory disease of the airways characterized by variable and recurring symptoms, reversible airflow obstruction, and bronchospasm. Symptoms include episodes of wheezing, coughing, chest tightness and shortness of breath and are often symptomatically diagnosed and treated with broncho-dilatators and/or steroids for inhalation.

Known risk factors for bronchial asthma are family history of asthma, allergies, respiratory infections [1], environmental pollutions (including dust mite [2] and air pollution [3]), tobacco smoking [4] and obesity [5]. Some studies also find female gender to be a risk factor [6].

The recent changes in attitude towards cannabis use, where the drug is perceived as almost harmless [7], and recent changes in legislation regulating its use, especially in the US, may increase the risk of asthma from increased cannabis use [8]. Greater awareness of the possible negative consequences of cannabis use would be prudent. Since cannabis, despite the development of novel ways of use, is most often smoked as marijuana by itself or as hashish together with tobacco, there is concern that its use might inflict respiratory consequences [9].

Cannabis users seem to have an increased risk of chronic bronchitis [10], reporting signs like coughing, sputum and wheezing, but no more shortness of breath [11–13]. A feared long-term negative consequence of chronic bronchitis is chronic obstructive pulmonary disorder (COPD) [14], but current research suggests that the use of cannabis does not increase the risk of COPD [15, 16].

Another consequence of cannabis smoking could be bronchial asthma. Three lines of research have been followed in this context. Firstly, some studies have investigated the potential acute bronchodilator effects of cannabis. Several older studies have shown a significant *positive* airway effect on bronchial asthma of cannabis administered in different ways to both healthy volunteers and asthmatic patients [10, 17–21]. Secondly, some cases have been observed where allergy to some components of cannabis seems to precipitate asthma [22, 23]. Thirdly, several larger population studies found an increase in symptoms of bronchial asthma in cannabis users: Several US cross-sectional health surveys have found more bronchial asthma among users of cannabis compared to others, even after controlling for age, gender, and tobacco use [13, 16, 24]. Moreover, three publications from the longitudinal Dunedin birth cohort study initially found an effect on asthma among all cannabis users, but when controlling for several confounders only found the association in women [25]. Results from the study also showed a positive effect on asthma from quitting cannabis [26]. Most population studies control for gender and tobacco use [13, 16, 24–27], some by analysing the effect only in non-users of tobacco [24]. Some even control for previous asthma [16, 25, 27], but few if any control for being overweight or for the presence of allergies. Overall the studies together suggest that there is an association of cannabis with bronchial asthma [28], with an overall effect a little less than for tobacco smokers [13]. Still, there are a limited number of studies investigating the relationship between cannabis use and bronchial asthma while controlling for a variety of potential covariates, and further studies are therefore needed [9]. We have found no studies with the prescriptions for asthma medication as outcome measure.

In Norway cannabis is mostly consumed as hashish, the resin of cannabis, prepared and mixed with tobacco and inhaled in cigarettes or joints. It is therefore important to control for tobacco smoking when investigating the possible effects of cannabis on the use of drugs for bronchial asthma.

In this longitudinal study we investigated the relationship between self-reported cannabis use and future filling of prescriptions for inhaled bronchodilators or steroids for the treatment of bronchial asthma, taking into consideration age, gender, weight, smoking and asthma and allergies.

## Methods

### Procedure and participants

This study is based on data from the Young in Norway Study, described in more detail elsewhere [29, 30]. A population-based sample of Norwegian adolescents was followed over a 13-year span with four data collections. The initial sample at the first time point (T_1_) was composed of 12,287 persons with a response rate of 97%. Only parts of the sample was invited for follow-up at later time points, and the cumulative response rate over all four data collection times for those who were eligible to be included at all data collection points was 69%. Participants were asked to give their consent to obtain information about them in various nationwide official registers such as the Norwegian Prescription Database (NorPD), and 90.0% consented to such linkage. In this study, we drew on the available data from 2602 individuals, 1145 males (44.0%) and 1457 females (56.0%). Survey data were collected at four times and mean age of the respondents at these data collection points was T_1_: 15.05 (SD = 1.96 in 1992), T_2_: 16.53 (1994), T_3_: 22.95 (1999) and T_4_: 28.48 years (2005–6), respectively.

Questionnaire data from the Young in Norway Study were linked to register data from the NorPD. Since 1 January 2004, all pharmacies in Norway are obliged, by law, to submit monthly electronic data on dispensed prescriptions to the Norwegian Institute of Public Health. The NorPD contains information on all prescription drugs, reimbursed or not, dispensed at Norwegian pharmacies to individual patients who live outside institutions [31]. The register contains information about all prescriptions, including the patients’ unique identifiers (encrypted), gender, age, date of dispensing and drug information, including brand name and anatomical therapeutic chemical (ATC) code [32]. The data from the Young in Norway Study and NorPD were linked by Statistics Norway as a third party ensuring the anonymity of the responders. The survey data from Young in Norway Study or NorPD were not visible for Statistics Norway during the linkage prosess.

### Measures

Cannabis use parameters were taken from the Young in Norway Study. Cannabis use was measured at T_4_. We categorized respondents into three groups according to their self-reported use of cannabis: those who had never used cannabis; those who had used cannabis at least once in their lifetime, but not in the last 12 months; and those who had used cannabis at least once during the last 12 months.

Gender, age, years of education and body mass index (BMI; kg/m^2^) were taken from reports at T_4_, while self-reported information on asthma (“Do you have asthma?” no/yes) and allergies (“Are you bothered by allergies?” no/yes) were taken from the earlier data collection at T_1_. Information on tobacco smoking habits were taken from T_4_, categorizing the responders into: those who had never smoked regularly; those who smoked regularly before, but not now; those who currently smoked sometimes, but not daily; and current daily smokers.

Information about all anti-asthma medication prescriptions between 2007 and 2015 were obtained through the NorPD, and we compared participants who did not obtain any prescriptions for these drugs in this time interval with participants who did. The interval between self-reported cannabis use and filling a prescription could thus vary from 1 to 9 years. The drugs studied were β_2_-adrenergic receptor agonists (ATC code R03A*) and glucocorticoids for inhalation (R03B*).

### Statistical analysis

Bivariate relationships between the explanatory variables and the outcome variable “prescriptions for anti-asthmatic drugs” were examined using chi square tests for categorical variables and Student’s T-tests for continuous variables. We also examined how our main explanatory variable “cannabis use” was related to other explanatory variables by using chi square for categorical variables and ANOVA for continuous variables. In a new set of analyses we performed binary logistic regressions with anti-asthmatic drug prescriptions as outcome, presenting firstly unadjusted odds ratios (OR) with 95% confidence intervals (95% CI), then a model adjusting for gender, age, earlier self-reported asthma and allergies, and a final model adjusting additionally for level of education, BMI and smoking habits. *P*-values of less than 0.05 were considered statistically significant, but mostly exact *p*-values are presented.

## Results

Women were prescribed anti-asthmatic drugs more often than men (*p* < 0.001), but there seemed to be no effect of age, years of education or BMI (Table 1). Those who reported at T_1_ to be bothered with asthma (*p* < 0.001) and allergies (*p* = 0.005) more often filled prescriptions for anti-ansthmatic drugs. Daily current tobacco smokers also filled prescriptions more often (*p* = 0.007), as did current users of cannabis (*p* = 0.009).

**Table 1:** Distribution of predictor variables according to having filled prescriptions for anti-asthma medication between 2007 and 2015

		No prescriptions		At least one prescription		Difference test Test statistics/*p*-value	
Gender							
Men	N (%)	1076	94.0	69	6.0		
Women	N (%)	1307	89.7	150	10.3	15.16	<.001
Age	Mean (SD)	28.49	1.96	28.41	2.21	0.60	.551
Education (in years)	Mean (SD)	14.96	2.17	14.88	2.11	0.50	.615
Body mass index	Mean (SD)	24.36	4.04	24.60	4.34	0.80	.423
Asthma at T_1_							
No asthma	N (%)	2155	92.3	179	7.7		
Asthma	N (%)	125	82.2	27	17.8	19.13	<.001
Allergies at T_1_							
No allergies	N (%)	1817	92.5	147	7.5		
Allergies	N (%)	463	88.7	59	11.3	7.91	.005
Smoking at T_4_							
Has never smoked	N (%)	1315	92.9	100	7.1		
Smoked daily before, but not now	N (%)	340	89.9	38	10.1		
Smokes sometimes, but not daily	N (%)	261	92.9	20	7.1		
Smokes daily	N (%)	457	88.4	60	11.6	12.10	.007
Cannabis use at T_4_							
Never used cannabis	N (%)	1569	92.0	137	8.0		
Has use cannabis before, but not last year	N (%)	483	93.2	35	6.8		
Used cannabis last year	N (%)	277	87.4	40	12.6	9.50	.009

^a^t-values for continous variables; χ^2^-values for categorical variables

Outcome measures are register data from the Norwegian Prescription Database and explanatory variables are taken from the longitudinal Young in Norway Study

Current users of cannabis were more often men (*p* < 0.001) and of young age (*p* < 0.001) (Table 2). Years of education was not related to use of cannabis, but BMI tended to be somewhat lower amongst cannabis users (*p* = 0.059). Neither self-reported asthma nor allergies at T_1_ were related to cannabis use, but current tobacco smokers, both daily and occasional, more often reported being current cannabis users (*p* < 0.001).

**Table 2:** Distribution of predictor variables according to use of cannabis at T_4_

		Never used cannabis		Has used cannabis before, but not last year		Has used cannabis last year		Difference test Test statistics^a^/*p*-value	
Gender									
Men	N (%)	682	60.7	247	22.0	194	17.3		
Women	N (%)	1024	72.2	271	19.1	123	8.7	52.03	< 0.001
Age	Mean (SD)	28.56	2.01	28.46	1.88	28.07	1.91	8.34	< 0.001
Education (in years)	Mean (SD)	14.92	2.14	15.03	2.20	15.05	2.26	0.75	0.471
Body mass index	Mean (SD)	24.49	4.06	24.33	4.18	23.90	3.70	2.83	0.059
Asthma at T1									
No asthma	N (%)	1530	67.0	462	20.2	292	12.8		
Asthma	N (%)	100	69.0	33	22.8	12	8.3	2.72	0.256
Allergies at T1									
No allergies	N (%)	1290	67.4	394	20.6	231	12.1		
Allergies	N (%)	340	66.1	101	19.6	73	14.2	1.74	0.419
Smoking at T4									
Has never smoked	N (%)	1132	81.9	175	12.7	75	5.4		
Smoked daily before, but not now	N (%)	208	56.4	121	32.8	40	10.8		
Smokes sometimes, but not daily	N (%)	135	48.4	72	25.8	72	25.8		
Smokes daily	N (%)	226	45.0	147	29.3	129	25.7	360.28	< 0.001

^a^F-values for continous variables; χ2-values for categorical variables

All data taken form the longitudinal Young in Norway Study

In binary logistic regressions with filling prescriptions for anti-asthmatic drugs as outcome, female gender, reported asthma and allergies at T_1_, current daily smoking, and cannabis use last year were all significantly associated with filling a prescription for asthma medications (Table 3, Unadjusted Model). In a model adjusting for gender, age, self-reported asthma and allergies at T_1_(Table 3, Model 1), results showed that females had a two-fold increased odds of filling such a prescription (*p* < 0.001), those with self-reported asthma at T_1_ had a 2.5 times increased odds of filling such a prescription (*p* < 0.001) and those currently using cannabis had a 2.1 times increased odds of filling a prescription for asthma medications (*p* = 0.028). In a model additionally adjusting for level of education, BMI and smoking habits (Table 3, Model 2), approximately the same values were found, other than self-reported allergies at T_1_ which emerged to be significantly associated with filling prescriptions for anti-asthmatic drugs (*p* = 0.025). We found no significant relationship between current daily smoking and filling prescriptions for anti-asthmatic drugs (OR 1.20; 95% CI: 0.78–1.85). The OR for filling a prescription for asthma medication among recent users of cannabis was 1.71 (95% CI: 1.06–2.77) in this final model.

**Table 3:** Logistic regression results with prescriptions of anti-asthma medication as outcome

	Reference (OR = 1)	Unadjusted			Model 1			Model 2		
		OR	95% CI	*p*	OR	95% CI	*p*	OR	95% CI	*p*
Female gender	Male	1.79	1.22–2.41	< 0.001	2.00	1.45–2.75	< 0.001	2.15	1.51–3.05	< 0.001
Age	Continuous	0.98	0.91–1.05	0.551	0.98	0.91–1.05	0.547	0.96	0.89–1.05	0.370
Education	Continuous	0.98	0.92–1.05	0.615				0.97	0.90–1.05	0.442
Body mass index	Continuous	1.01	0.98–1.05	0.423				1.04	1.00–1.08	0.050
Asthma at T1	No asthma	2.60	1.67–4.05	< 0.001	2.48	1.52–4.03	< 0.001	2.49	1.50–4.13	< 0.001
Allergies at T1	No allergies	1.58	1.15–2.17	0.005	1.38	0.98–1.95	0.067	1.51	1.05–2.17	0.025
Smoking at T4	Never smoked									
Smoked daily before, but not now		1.47	0.99–2.18	0.054				1.15	0.73–1.83	0.540
Smokes sometimes, but not daily		1.01	0.61–1.66	0.976				0.88	0.51–1.56	0.678
Smokes daily		1.73	1.23–2.42	0.002				1.20	0.78–1.85	0.414
Cannabis use at T4	Never used cannabis									
Used cannabis before, but not last year		0.83	0.57–1.22	0.342	0.85	0.57–1.27	0.423	0.82	0.53–1.28	0.378
Used cannabis last year		1.65	1.14–2.41	0.009	2.05	1.38–3.05	< 0.001	1.71	1.06–2.77	0.028

Note: OR = odds ratio; 95% CI = 95% confidence interval of OR

Data on prescriptions are taken from the Norwegian Prescription Database and and explanatory variables are taken from the longitudinal Young in Norway Study. Model 1 includes adjustment for gender, age, self-reported asthma or allergies in addition to cannabis smoking. Model 2 includes additional adjustment for education, BMI and smoking

We also performed a binary logistic regression stratifying the material according current daily smoking (those currently smoking daily and all others analyzed separately). This analysis did not change the outcome of the binary logistic regression substantially, even though the association between cannabis use and prescription of asthma medication did not reach significance among the current daily smokers (*p* > 0.05; data not shown in the table), probably because to the size of the group of current smokers was too small (*N* = 457).

## Discussion

This study combined survey data and data from a national prescription registry to demonstrate that the filling of presciptions for asthma medications was related to current, but not former, cannabis use. The odds for filling a prescription increased two-fold for current cannabis users compared to those who had never used cannabis and this increased odds withstood adjustment for all other relevant risk factors, such as female gender, self-reported asthma and allergies in adolescence, and even daily smoking in a comprehensive regression model.

The use, in our study, of prescription of asthma medication as outcome measure is novel, but our finding is in line with several studies indicating a negative effect of cannabis use on respiratory function and the precipitation of asthma [13, 16, 24–27]. We found an increase of approximately 70% in the prescription of asthma medication among cannabis users. This increase is comparable to other studies which have found similar levels of increased risk [13, 16, 26, 27].

The validity of the study design and the findings was strengthened by fact that the study identified that participants’ self-report of having asthma at an earlier time point was, in itself, a risk factor for filling a prescription for asthma medications. Study findings are also in accordance with previous studies by showing thatpreviously identified risk factors such as female gender [6], tobacco smoking [4] and at a trend level BMI [5] increased the odds of asthma medication being prescriped. Controlling for these factors only marginally changed the relationship between cannabis use and filling a prescription for asthma medication. We cannot ultimately rule out the possibility of residual confounding. We were, however, able to control for having allergies, a known risk factor for asthma [1], but this still did not change the impact of current cannabis use on asthma. We have found no other studies investigating the relationship beween cannabis use and asthma medication that have been able to adjust for self-reported allergies.

Our study also indicated no effect of former (but not current) cannabis use on asthma medication prescription. Former use could indicate people who have only tried cannabis, an exposure less likely to have negative health effects. As we lack measures on the amount of cannabis used, this finding should be interpreted cautiously, but it may be in line with earlier suggestions that quitting cannabis is beneficial for lung function and reduces asthma symptoms [26].

Our study cannot completely rule out the possibility of reversed causality, i.e. that patients with asthma use cannabis to relieve symptoms. Some studies do show that cannabis may relieve symptoms of airway obstruction and asthma [10, 17–21]. However, our results show that asthma in adolescent years (at T_1_) was not related to cannabis use. Moreover, we did adjust for asthma at an earlier time point in our study. It is still possible that people are using cannabis to alleviate asthma symptoms, without a formal diagnosis of asthma or awareness of having the condition.

The study had a sufficiently large cohort of young adults with enough exposure to cannabis in order to detect potential negative effects of cannabis use. The national coverage of NorPD ensured complete data for prescriptions filled. We do not know, however, if prescribed drugs were in fact used, as we have no information of secondary non-compliance. Furthermore, filling a prescription for asthma medication is not the same as a diagnosis of asthma. A prescription is only a proxy for the disease. Investigations show that asthma may be undertreated [33], and this may lead to an under-estimation of a diagnosis of asthma. We have no information regarding whether such under-treatment should be more or less severe among cannabis users, and do not know if this has introduced a bias. Furthermore, the responders were followed for a sufficient time to pick up on negative health effects, as we followed the respondents’ prescription records up to 9 years after the last questionnaire. However, the study is limited by not providing information about how much cannabis participants had used or if they were using cannabis in the whole time period.

## Conclusions

The study strengthens earlier findings and suggests that current use of cannabis is a risk factor for precipitating asthma, even when other known risk factors for asthma are taken in to consideration. No previous studies have used asthma medication as an outcome. These findings are important in the light of the changes in legislation being considered in many countries. Those who opt taking cannabis may need to find alternatives to smoking it. The ongoing measurement of respiratory function amongst cannabis users is advisable.

## Abbreviations

ANOVA: Analysis of variance
ATC: Anatomical-Therapeutic-Chemical
BMI: Body Mass Index
CI: Confidence Interval
COPD: Chronic Obstructive Pulmonary Disease
NorPD: Norwegian Prescription Database

## References

[1] Bonnelykke K, Vissing NH, Sevelsted A, Johnston SL, Bisgaard H (2015). Association between respiratory infections in early life and later asthma is independent of virus type. J Allergy Clin Immunol.

[2] Fernandez-Caldas E, Puerta L, Caraballo L (2014). Mites and allergy. Chem Immunol Allergy.

[3] Guarnieri M, Balmes JR (2014). Outdoor air pollution and asthma. Lancet.

[4] Sands MF (2014). Smoking and asthma: never the twain should meet. Ann Allergy Asthma Immunol.

[5] Boulet LP (2013). Asthma and obesity. Clin Exp Allergy.

[6] Leynaert B, Sunyer J, Garcia-Esteban R, Svanes C, Jarvis D, Cerveri I (2012). Gender differences in prevalence, diagnosis and incidence of allergic and non-allergic asthma: a population-based cohort. Thorax.

[7] Golick J (2016). Shifting the paradigm: adolescent Cannabis abuse and the need for early intervention. J Psychoactive Drugs.

[8] Hall W, Weier M (2015). Assessing the public health impacts of legalizing recreational cannabis use in the USA. Clin Pharmacol Ther.

[9] Gates P, Jaffe A, Copeland J (2014). Cannabis smoking and respiratory health: consideration of the literature. Respirology.

[10] Tetrault JM, Crothers K, Moore BA, Mehra R, Concato J, Fiellin DA (2007). Effects of marijuana smoking on pulmonary function and respiratory complications: a systematic review. Arch Intern Med.

[11] Aldington S, Williams M, Nowitz M, Weatherall M, Pritchard A, McNaughton A (2007). Effects of cannabis on pulmonary structure, function and symptoms. Thorax.

[12] Bloom JW, Kaltenborn WT, Paoletti P, Camilli A, Lebowitz MD (1987). Respiratory effects of non-tobacco cigarettes. Br Med J (Clin Res Ed).

[13] Moore BA, Augustson EM, Moser RP, Budney AJ (2005). Respiratory effects of marijuana and tobacco use in a U.S. sample. J Gen Intern Med.

[14] Pelkonen M (2008). Smoking: relationship to chronic bronchitis, chronic obstructive pulmonary disease and mortality. Curr Opin Pulm Med.

[15] Tashkin DP (2015). Does marijuana pose risks for chronic airflow obstruction?. Ann Am Thorac Soc.

[16] Kempker JA, Honig EG, Martin GS (2015). The effects of marijuana exposure on expiratory airflow. A study of adults who participated in the U.S. National Health and nutrition examination study. Ann Am Thorac Soc.

[17] Tashkin DP, Shapiro BJ, Frank IM (1974). Acute effects of smoked marijuana and oral delta9-tetrahydrocannabinol on specific airway conductance in asthmatic subjects. Am Rev Respir Dis.

[18] Tashkin DP, Shapiro BJ, Lee YE, Harper CE (1975). Effects of smoked marijuana in experimentally induced asthma. Am Rev Respir Dis.

[19] Tashkin DP, Reiss S, Shapiro BJ, Calvarese B, Olsen JL, Lodge JW (1977). Bronchial effects of aerosolized delta 9-tetrahydrocannabinol in healthy and asthmatic subjects. Am Rev Respir Dis.

[20] Abboud RT, Sanders HD (1976). Effect of oral administration of delta-tetrahydrocannabinol on airway mechanics in normal and asthmatic subjects. Chest.

[21] Williams SJ, Hartley JP, Graham JD (1976). Bronchodilator effect of delta1-tetrahydrocannabinol administered by aerosol of asthmatic patients. Thorax.

[22] Vidal C, Fuente R, Iglesias A, Saez A (1991). Bronchial asthma due to Cannabis sativa seed. Allergy.

[23] Kumar R, Gupta N (2013). A case of bronchial asthma and allergic rhinitis exacerbated during Cannabis pollination and subsequently controlled by subcutaneous immunotherapy. Indian J Allergy Asthma Immunol.

[24] Charilaou P, Agnihotri K, Garcia P, Badheka A, Frenia D, Yegneswaran B. Trends of Cannabis use disorder in the inpatient: 2002 to 2011. Am J Med 2017; 130: 678–87.e7. 10.1016/j.amjmed.2016.12.035 28161344

[25] Robinson PD, King GG, Sears MR, Hong CY, Hancox RJ. Determinants of peripheral airway function in adults with and without asthma. Respirology. 2017. 10.1111/resp.13045. 10.1111/resp.13045 28397998

[26] Hancox RJ, Shin HH, Gray AR, Poulton R, Sears MR (2015). Effects of quitting cannabis on respiratory symptoms. Eur Respir J.

[27] Taylor DR, Poulton R, Moffitt TE, Ramankutty P, Sears MR (2000). The respiratory effects of cannabis dependence in young adults. Addiction.

[28] Tashkin DP (2014). Increasing cannabis use: what we still need to know about its effects on the lung. Respirology.

[29] von Soest T, Bramness JG, Pedersen W, Wichstrom L (2012). The relationship between socio-economic status and antidepressant prescription: a longitudinal survey and register study of young adults. Epidemiol Psychiatr Sci.

[30] von Soest T, Wichstrom L, Kvalem IL (2016). The development of global and domain-specific self-esteem from age 13 to 31. J Pers Soc Psychol.

[31] Furu K (2008). Establisment of the nationwide Norwegian prescription databese (NorPD) – new opportunities for research in pharmacoepidemiology in Norway. Nor Epidemiol.

[32] WHO Collaborating Centre for Drug Statistics Methodology (2005). Guidelines for ATC classification and DDD assignment.

[33] Bollinger ME, Mudd KE, Boldt A, Hsu VD, Tsoukleris MG, Butz AM (2013). Prescription fill patterns in underserved children with asthma receiving subspecialty care. Ann Allergy Asthma Immunol.

